# Supplementary material for: Network-based machine learning in colorectal and bladder organoid models predicts anti-cancer drug efficacy in patients
Source: Nat Commun. 2020 Oct 30;11:5485. doi: 10.1038/s41467-020-19313-8 (PMC7599252; doi:10.1038/s41467-020-19313-8)
Supplement: Supplementary file 1 — Supplementary Information [file 41467_2020_19313_MOESM1_ESM.pdf]

Supplementary Information

Title: Network-based machine-learning in colorectal and bladder organoid models predicts anti-cancer drug efficacy in patients

To whom correspondence should be addressed:

Sanguk Kim, Ph.D.

Department of Life Sciences, Pohang University of Science and Technology, Pohang 790-784, Korea; E-mail: sukim@postech.ac.kr; Tel: +82-54-279-2348; Fax: +82-54-279-2199

Kunyoo Shin, Ph.D.

Department of Life Sciences, Pohang University of Science and Technology, Pohang 790-784, Korea; E-mail: kunyoos@postech.ac.kr;

Table of contents

Supplementary Information file contains 18 Supplementary Figures and 4 Supplementary Tables

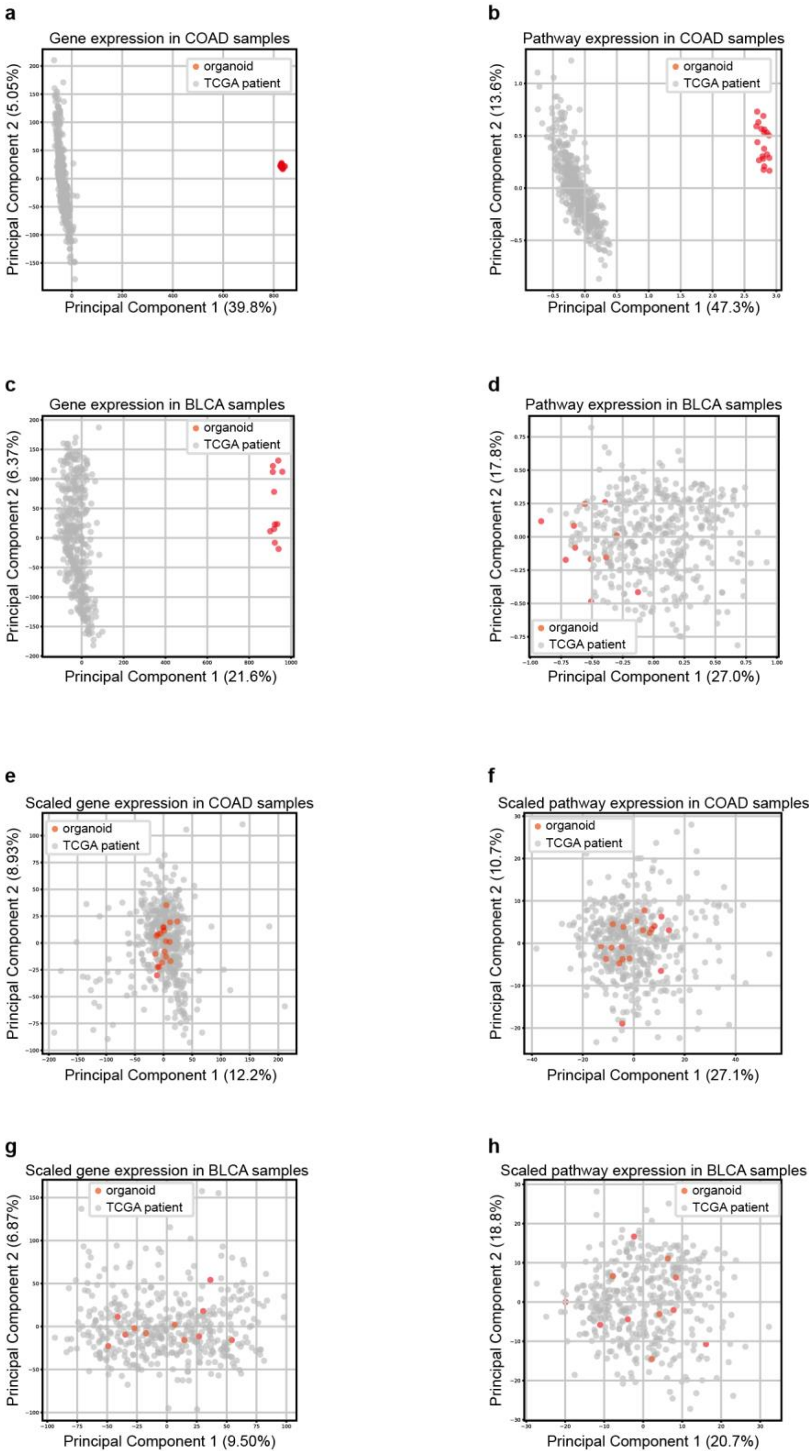

Supplementary Fig. 1. Removal of batch effects across transcriptomic data of The Cancer Genome Atlas (TCGA) patients and three-dimensional organoid models. Principal component analysis (PCA) was plotted against the two highest principal components. Gene expression- and pathway expression-based PCA plots for colorectal and bladder cancer samples

are plotted in (**a**)–(**d**). Scaled gene and pathway expression levels (see Methods) based PCA plots for colorectal and bladder cancer samples are plotted in (**e**)–(**h**). Red and gray dots indicate organoid and TCGA patient samples, respectively. The explained variance ratio for each principal component is shown inside the parentheses. COAD, colorectal cancer; BLCA, bladder cancer.

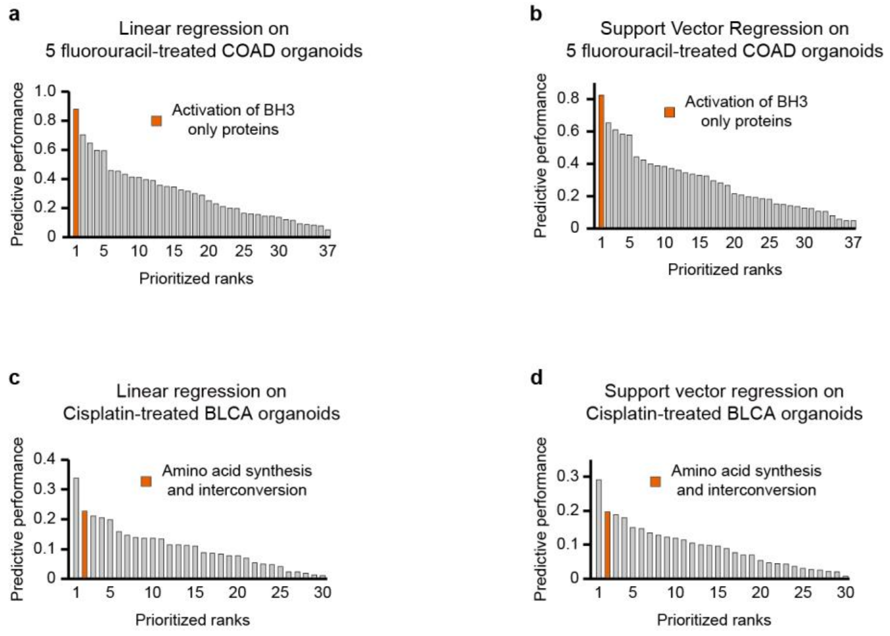

Supplementary Fig. 2. Predictive performances of proximal pathways against linear regression and support vector regression. Predictive performances of pathways in 5-fluorouracil-treated colorectal cancer (COAD) organoid models using linear regression (**a**) and support vector regression (**b**). Predictive performances of pathways in cisplatin-treated bladder cancer (BLCA) organoid models using linear regression (**c**) and support vector regression (**d**). Selected biomarkers are shown in orange.

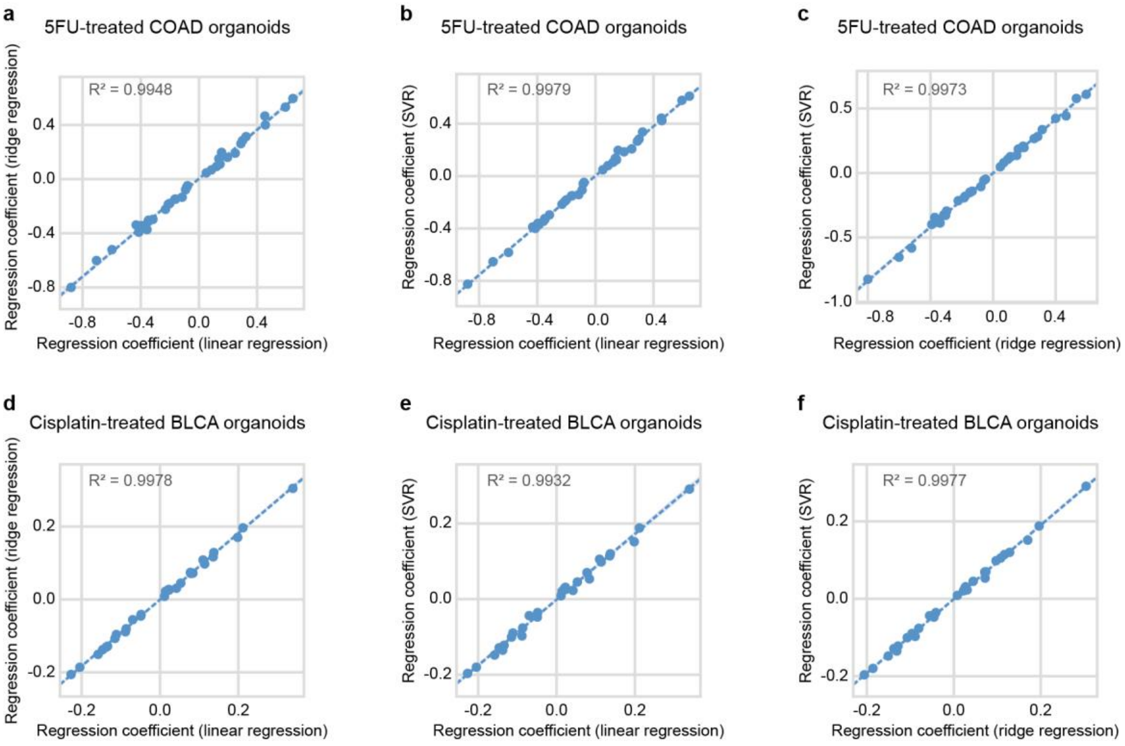

Supplementary Fig. 3. Comparison of regression coefficients between various machine-learning algorithms. Various machine-learning models were trained using the transcriptomes of proximal pathways and drug responses (IC_{50} values) from organoid models. Correlation of regression coefficients between machine-learning algorithms in (**a**)–(**c**) for 5-fluorouracil-treated colorectal cancer organoids and in (**d**)–(**e**) for cisplatin-treated bladder cancer organoids. The coefficient of determination is given as R^2. SVR, support vector regression. COAD, colorectal cancer; BLCA, bladder cancer.

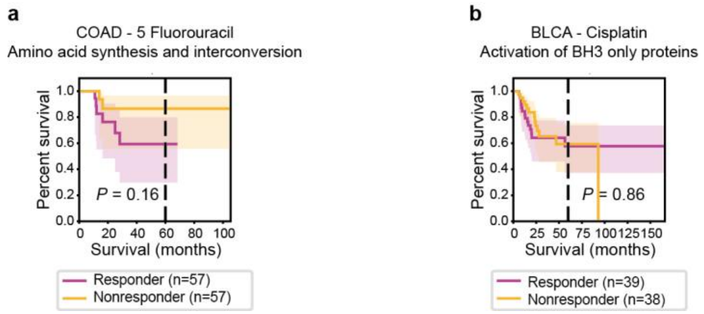

Supplementary Fig. 4. Identification of biomarkers across drug and cancer types. (**a**) The cisplatin biomarker in bladder cancer ("amino acid synthesis and interconversion" pathway) was used to classify 5-fluorouracil-treated colorectal cancer patients. Statistical significance was measured using Kaplan-Meier survival curves and two-sided log-rank tests. (**b**) The 5-fluorouracil biomarker in colorectal cancer ("activation of BH3-only proteins" pathway) was used to classify cisplatin-treated bladder cancer patients. Statistical significance was measured using Kaplan-Meier survival curves and two-sided log-rank tests. COAD, colorectal cancer; BLCA, bladder cancer.

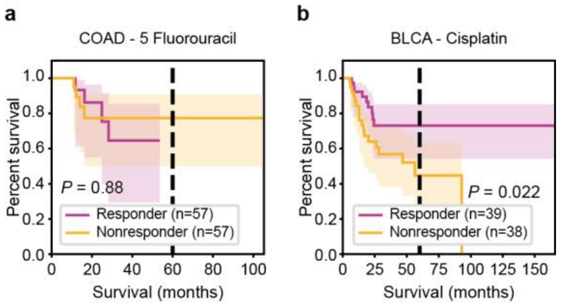

Supplementary Fig. 5. Prediction of drug response in cancer patients using the two pathways with the highest predictive performances in organoid models. Drug response prediction for (**a**) 5-fluorouracil-treated colorectal and (**b**) cisplatin-treated bladder cancer patients. Statistical significance was measured using Kaplan-Meier survival curves and two-sided log-rank tests. COAD, colorectal cancer; BLCA, bladder cancer.

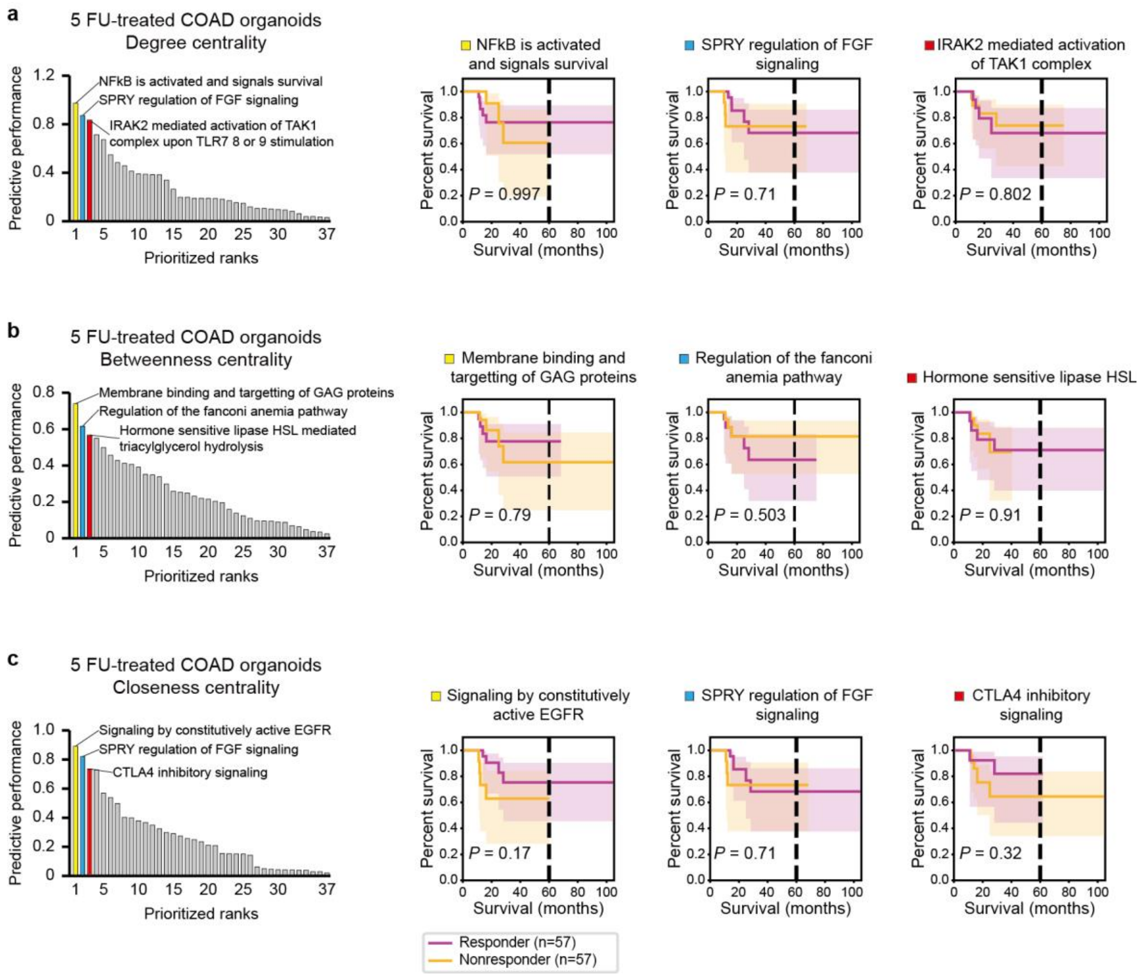

Supplementary Fig. 6. Prediction of drug response in 5-fluorouracil-treated colorectal cancer patients using central pathways to train colorectal cancer organoid models. Predictive performances of the top central pathways measured by (**a**) degree, (**b**) betweenness, and (**c**) closeness centrality. Kaplan-Meier survival plot for the top first (yellow), second (blue), and third (red) drug response predictive pathways. The average centrality scores of pathway genes were used to quantify pathway centrality. Statistical significance was measured using Kaplan-Meier survival curves and two-sided log-rank tests. COAD, colorectal cancer; BLCA, bladder cancer.

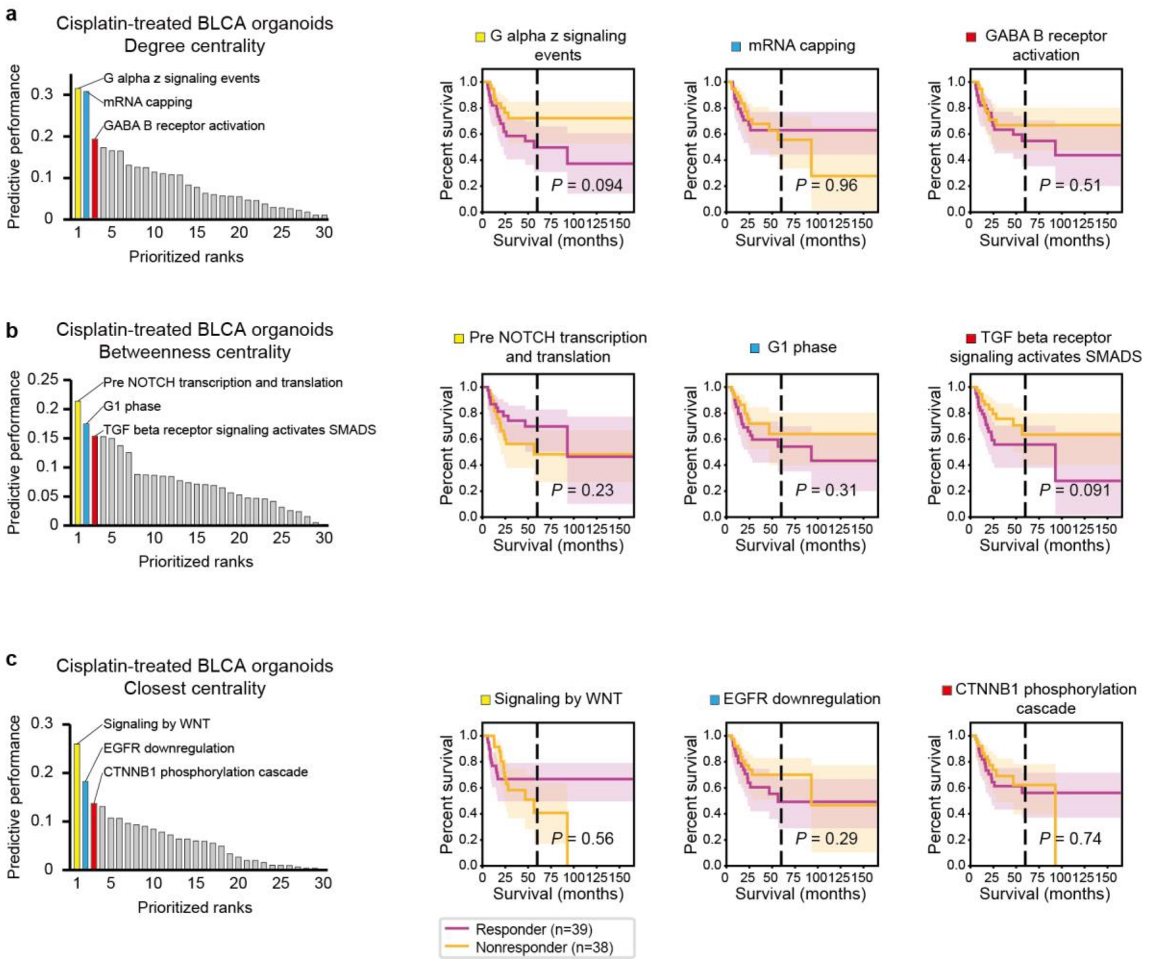

Supplementary Fig. 7. Prediction of drug response in cisplatin-treated bladder cancer patients using central pathways to train bladder cancer organoid models. Predictive performances of the top central pathways measured by (**a**) degree, (**b**) betweenness, and (**c**) closeness centrality. Kaplan-Meier survival plot for the top first (yellow), second (blue), and third (red) drug response predictive pathways. The average centrality scores of pathway genes were used to quantify pathway centrality. Statistical significance was measured using Kaplan-Meier survival curves and two-sided log-rank tests. COAD, colorectal cancer; BLCA, bladder cancer.

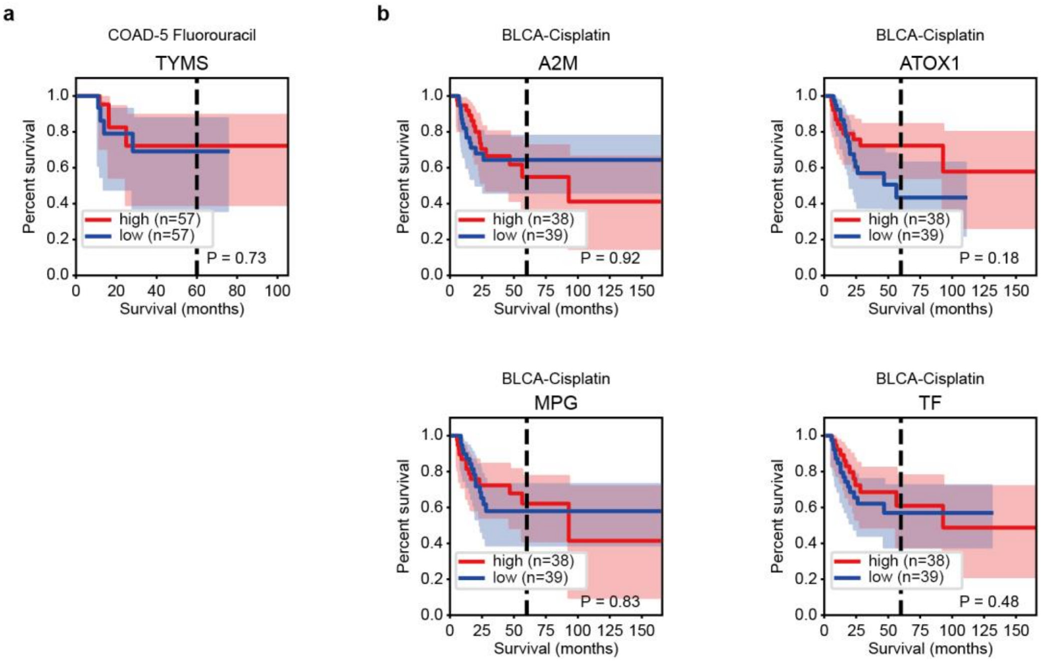

Supplementary Fig. 8. Survival analysis using the expression levels of drug target genes. (**a**) Kaplan-Meier plot for 5-fluorouracil-treated colorectal cancer patients. The gene expression profile of the 5-fluorouracil target, thymidylate synthetase (*TYMS*), was used to classify patients as responders or non-responders. The median expression level was used to stratify the patients into the two groups. Groups with high and low *TYMS* gene expression levels are shown in red and blue, respectively. *P*-values were calculated using two-sided log-rank test. *P*-values < 0.05 were considered significant. (**b**) Kaplan-Meier plots for cisplatin-treated bladder cancer patients. Cisplatin targets, alpha-2 macroglobulin (*A2M*), antioxidant 1 copper chaperone (*ATOX1*), N-methylpurine DNA glycosylase (*MPG*), and serotransferrin (*TF*), were independently used to classify patients. *P*-values were calculated using two-sided log-rank test. *P*-values < 0.05 were considered significant. The dashed line corresponds to the 5-year survival time (60 months). COAD, colorectal cancer; BLCA, bladder cancer.

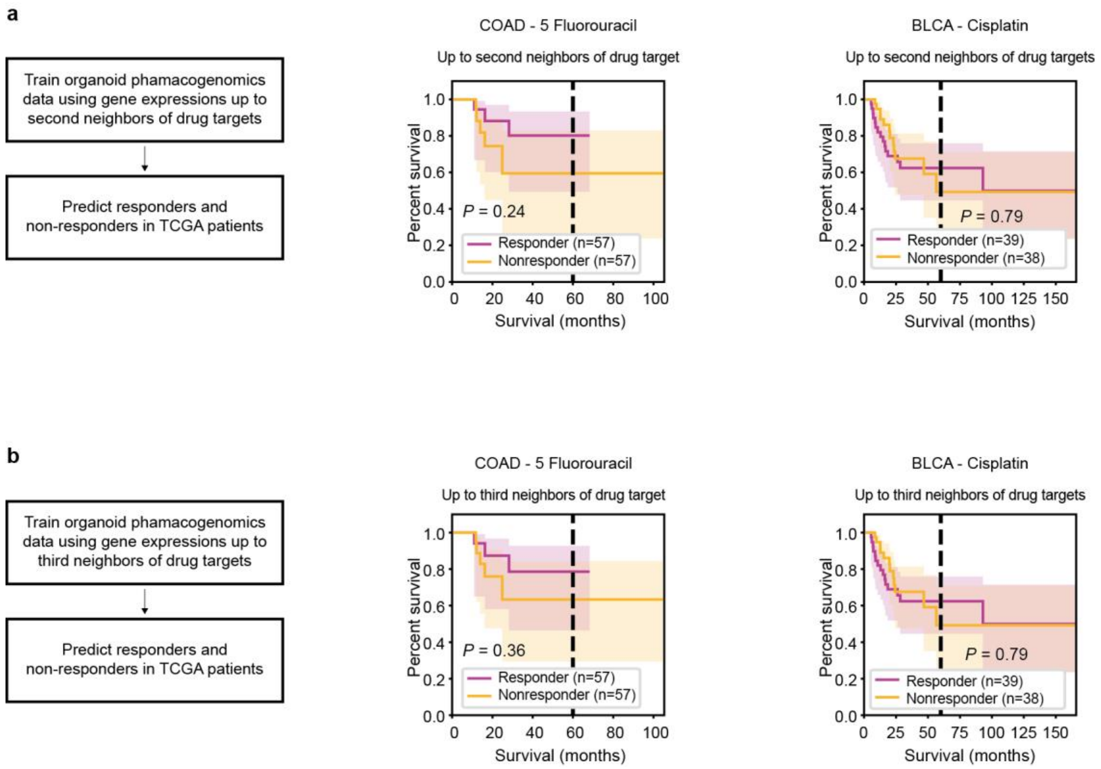

Supplementary Fig. 9. Survival analysis using the expression levels of drug target neighbor genes. Kaplan-Meier plot for genes up to the second (**a**) and the third neighbors of drug targets (**b**). Selected genes were used to train organoid pharmacogenomic data and predict responders and non-responders in 5-fluorouracil-treated colorectal and cisplatin-treated bladder cancer patients. The median of the predicted IC_{50} values was used to classify responders (purple) and non-responders (yellow). The two-sided log-rank test was used to measure the statistical significance of differences in overall survival between the two groups. COAD, colorectal cancer; BLCA, bladder cancer.

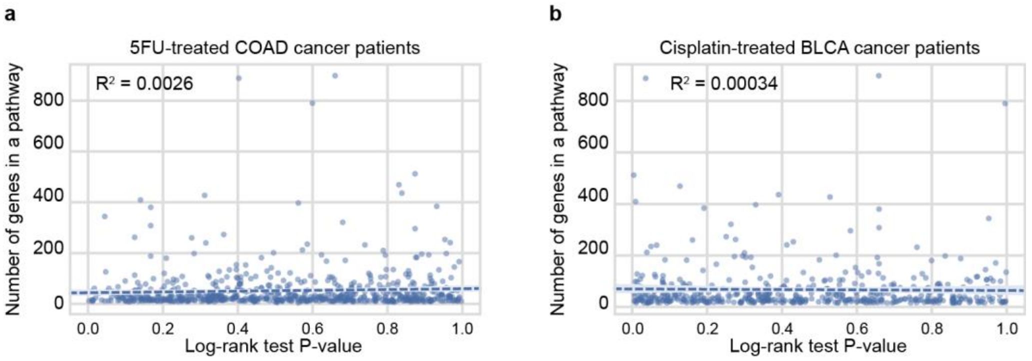

Supplementary Fig. 10. Correlation between the number of genes in a pathway and its predictive performance in cancer patient drug responses. Correlation between the number of genes in a pathway and drug response prediction in (**a**) 5-fluorouracil-treated colorectal and (**b**) cisplatin-treated bladder cancer patients. The coefficient of determination is given as R^2. Patients were divided into two groups according to the median pathway expression levels. COAD, colorectal cancer; BLCA, bladder cancer.

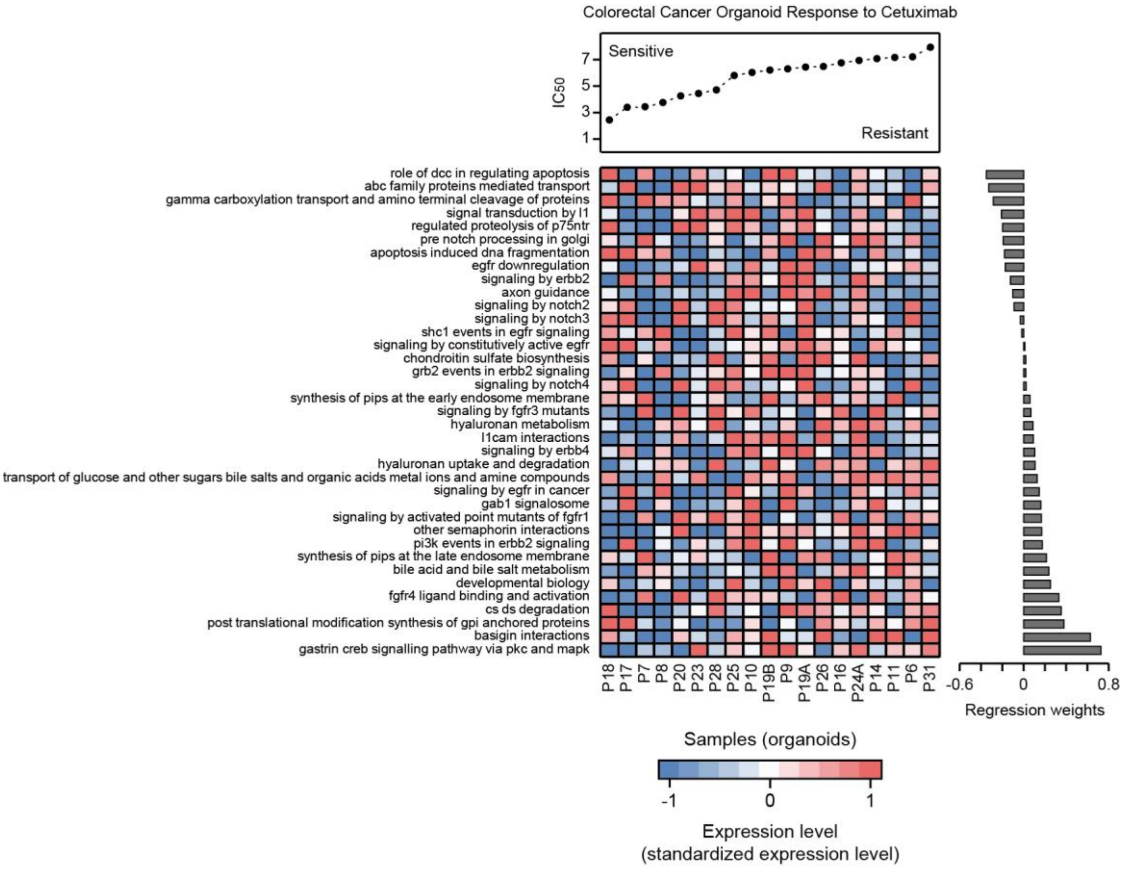

Supplementary Fig. 11. Predictive performances of proximal pathways for cetuximab response in colorectal cancer organoid models. Ridge regression for the expression profiles of the proximal pathways of cetuximab responses and drug responses (IC_{50}) from colorectal organoid data. Pathways are shown in each row of the heatmap, whereas organoid sample IDs are shown in each column. Samples (n = 19) are sorted from the most sensitive (left) to the most resistant (right) to cetuximab. Ridge regression coefficients are shown as regression weights.

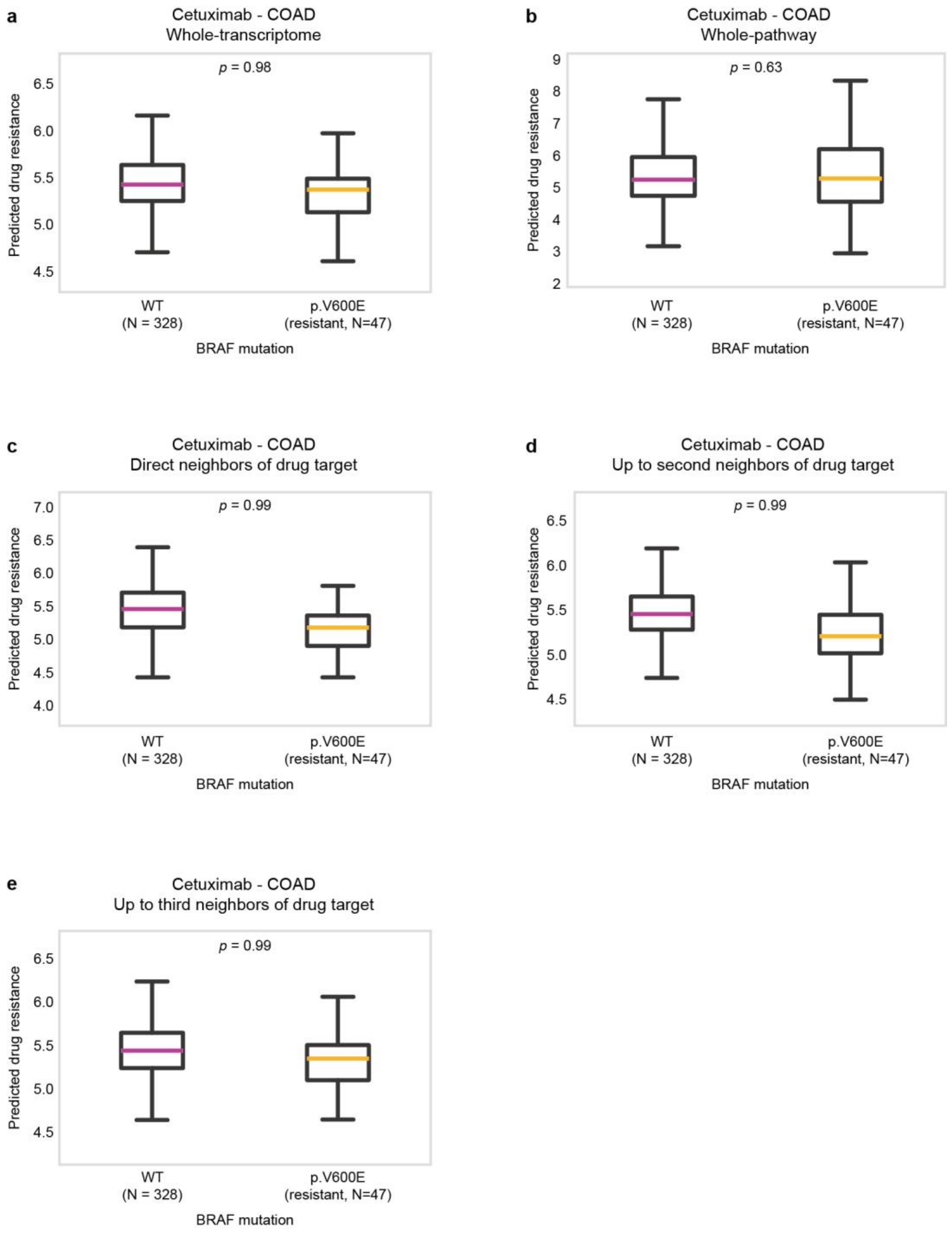

Supplementary Fig. 12. Association of predicted cetuximab resistance with known biomarkers in colorectal cancer using network-free or module-free models. Prediction of drug resistance using (**a**) whole-transcriptome profiles, (**b**) whole-pathway profiles, (**c**) direct neighbors of drug targets, (**d**) up to the second neighbors, and (**e**) up to the third neighbors as input features for the machine-learning models. Machine-learning models were trained using cetuximab sensitivity in colorectal cancer (COAD) organoid models. Statistical significance

was measured using a one-sided Mann-Whitney U test. Patient numbers are shown inside the parentheses. Boxplot displays median value, interquartile range (IQR) as bounds of box and whiskers extending to $\text{upper/lower quartile} \pm \text{IQR} \times 1.5$.

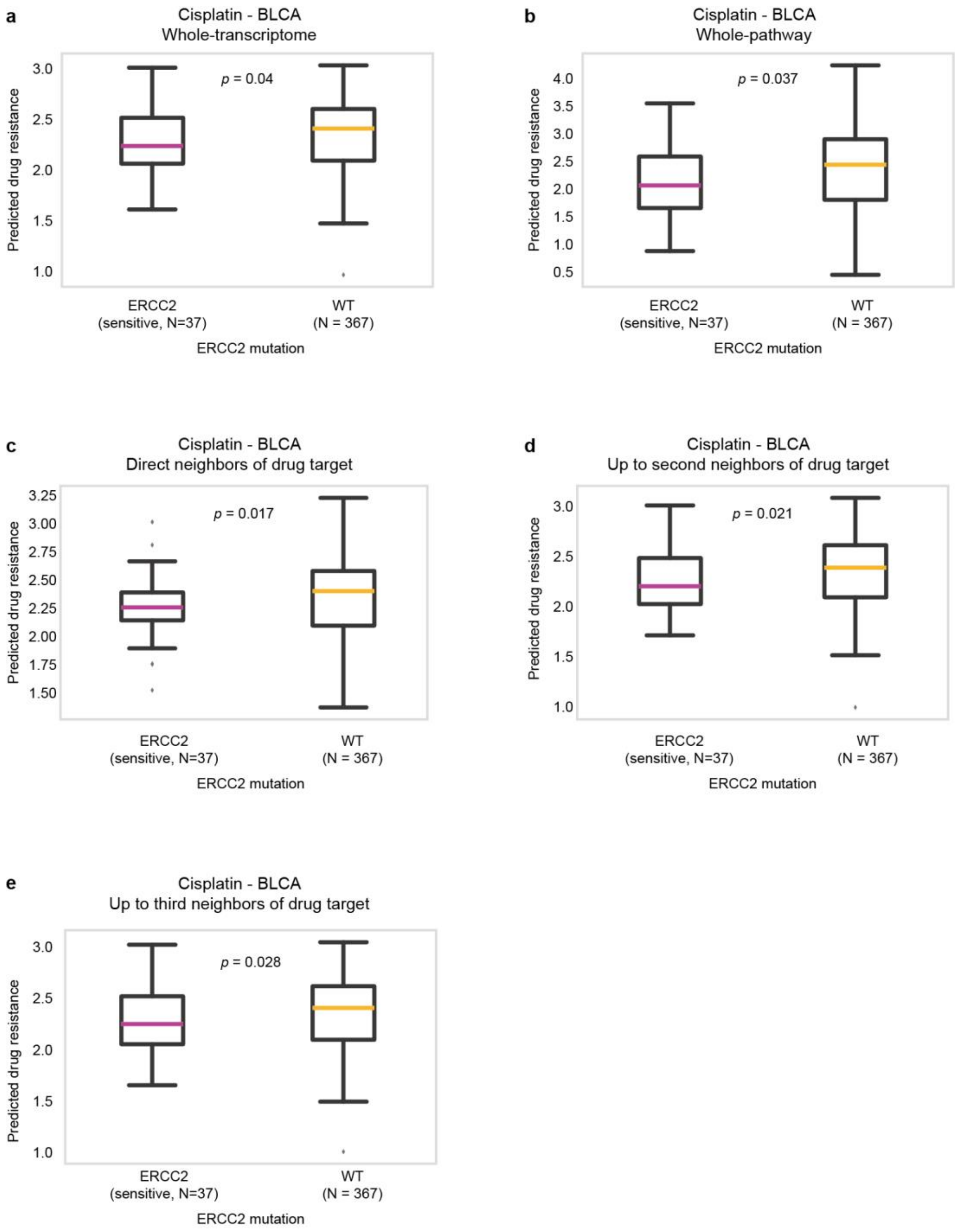

Supplementary Fig. 13. Association of predicted cisplatin resistance with known biomarkers in bladder cancer using network-free or module-free models. Prediction of drug resistance using (**a**) whole-transcriptome profiles, (**b**) whole-pathway profiles, (**c**) direct neighbors of drug targets, (**d**) up to the second neighbors, and (**e**) up to the third neighbors as input features for the machine-learning models. Machine-learning models were trained using cisplatin sensitivity in bladder cancer (BLCA) organoid models. Statistical significance was

measured using a one-sided Mann-Whitney U test. Patient numbers are shown inside the parentheses. Boxplot displays median value, interquartile range (IQR) as bounds of box and whiskers extending to $\text{upper/lower quartile} \pm \text{IQR} \times 1.5$.

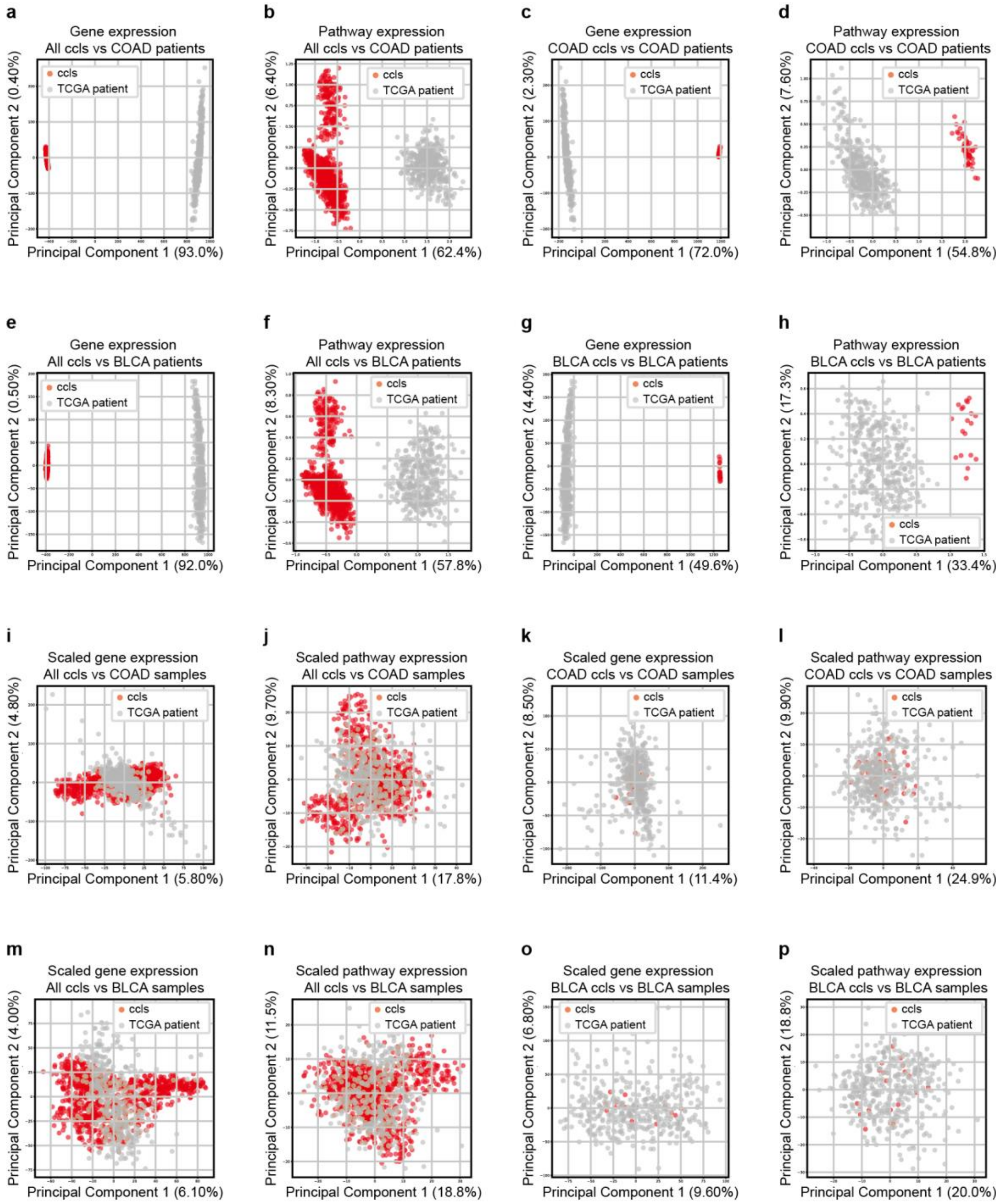

Supplementary Fig. 14. Removal of batch effects across transcriptomic data of The Cancer Genome Atlas (TCGA) patients and Genomics of Drug Sensitivity in Cancer (GDSC) cancer cell lines. Principal component analysis (PCA) was plotted against the two highest principal components. Gene expression- and pathway expression-based PCA plots for colorectal and bladder cancer samples are plotted in (**a**)–(**h**). Scaled gene and pathway expression levels (see Methods) based on the PCA plots for colorectal and bladder cancer

samples are plotted in (**i**)–(**p**). Red and gray dots indicate GDSC cancer cell lines and TCGA patient samples, respectively. The explained variance ratio for each principal component is shown inside the parentheses. COAD, colorectal cancer; BLCA, bladder cancer; CCL, cancer cell line.

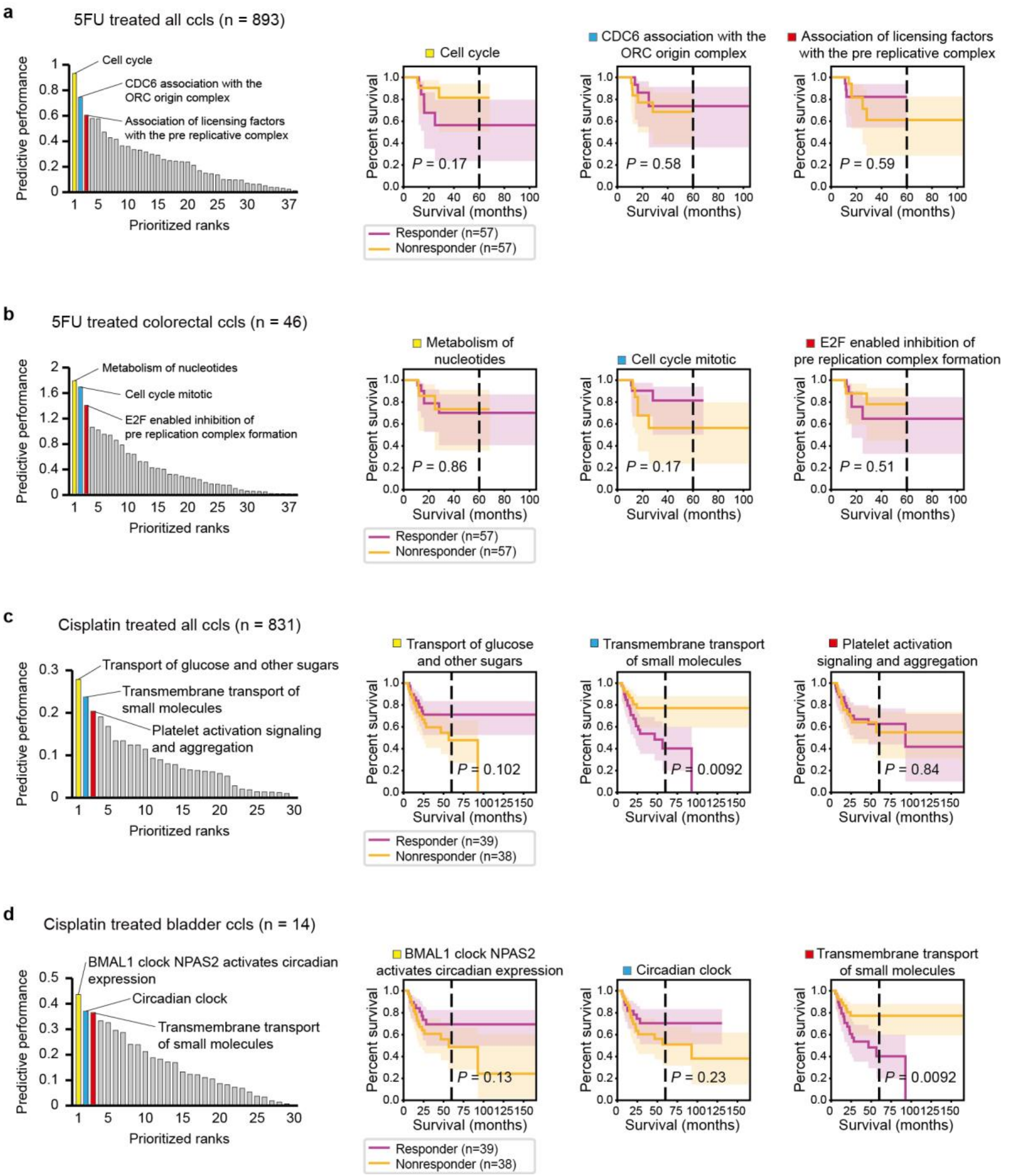

Supplementary Fig. 15. Drug response predictions of 5-fluorouracil-treated colorectal and cisplatin-treated cancer patients using the network-based approach in cancer cell lines from the Genomics of Drug Sensitivity in Cancer (GDSC) database. The drug response prediction of 5-fluorouracil in colorectal cancer patients using (**a**) all cancer cell lines (n = 893) and (**b**) colorectal cancer cell lines (n = 46). The drug response prediction of cisplatin in bladder cancer patients using (**c**) all cancer cell lines (n = 831) and (**d**) bladder cancer cell

lines (n = 14). *P*-values were calculated using two-sided log-rank test. ccls, cancer cell lines.

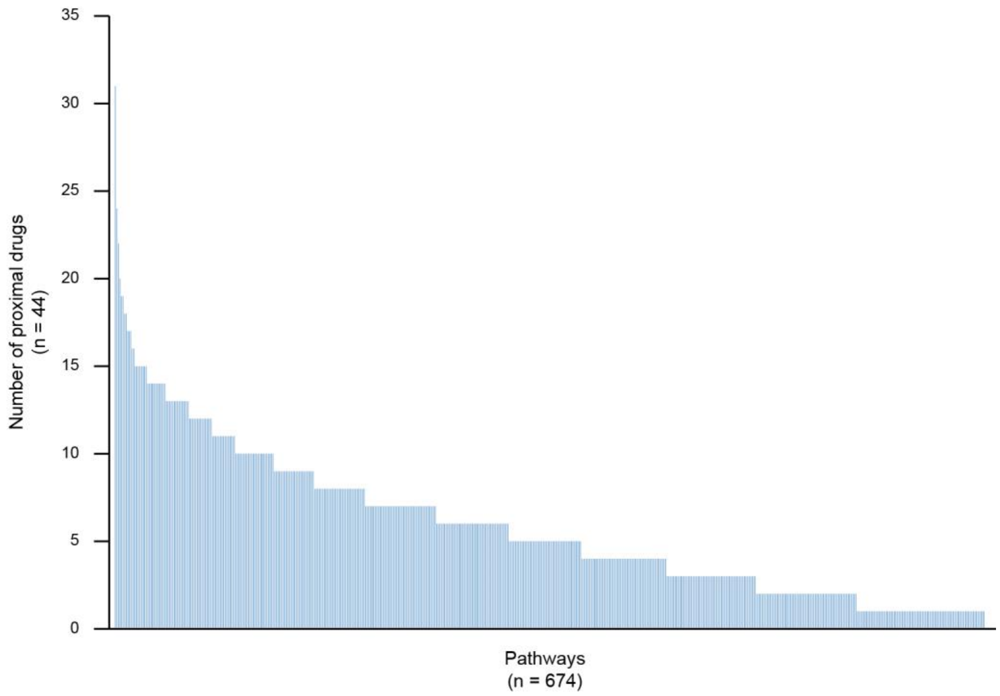

Supplementary Fig. 16. Number of proximal pathways with the drug targets of 44 drugs.

The number of proximal pathways (z-score $\leq$ -1.2816) for each drug (44 drugs total) is shown. The total numbers of drugs and pathways are shown inside the parentheses. Pathways that are not proximal to any of the 44 drugs are not displayed (n = 111 pathways).

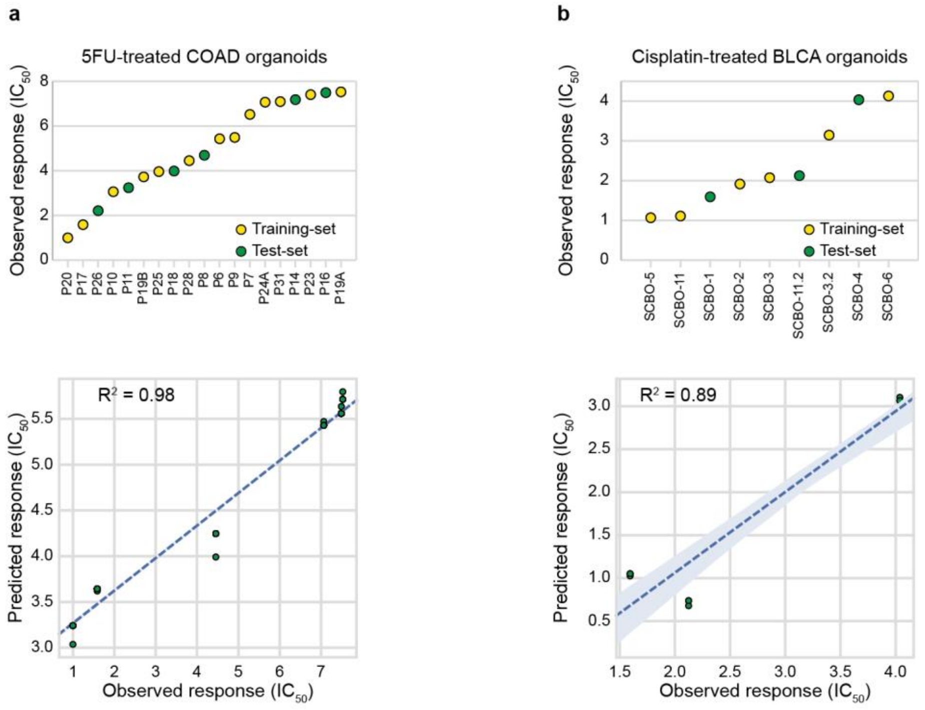

Supplementary Fig. 17. Prediction of drug response in a split organoid dataset. Organoid samples were split into training (60%), validation (10%), and test (30%) sets. Prediction performance was measured by comparing the observed and predicted drug responses in (**a**) 5-fluorouracil-treated colorectal and (**b**) cisplatin-treated bladder cancer organoids. Samples used for training and test sets are indicated as yellow and green dots, respectively. Linear regression lines are fitted to the observed and predicted responses and are displayed in dotted lines. Scatter plot shows the 95% confidence interval. COAD, colorectal cancer; BLCA, bladder cancer.

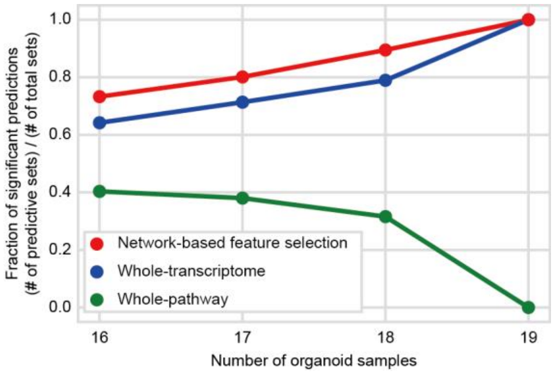

Supplementary Fig. 18. Robustness of network-based machine-learning predictions in recovering BRAF^{V600E} mutation-based Cetuximab resistance in colorectal cancer. Testing of BRAF^{V600E} mutation-based cetuximab resistance to determine if it can be recovered from a smaller number of colorectal cancer organoid models. Network-based machine-learning model or whole-transcriptome-based and whole-pathway-based machine-learning models are depicted in red or blue and green, respectively. Predictions were tested for 969, 171 and 19 non-identical combinations of samples for 16, 17 and 18 number of organoid samples, respectively.

Variable		Cases	Predicted Response		
			Responder	NonResponder	P-value
Age (years)					
	<60	53	30	23	0.26
	≥60	61	27	34	
Gender					
	Male	62	27	35	0.19
	Female	52	30	22	
T stage					
	T1-T2	7	2	5	0.44
	T3-T4	107	55	52	
Lymph node stage					
	N0	27	16	11	0.44
	N1	52	26	26	
	N2	35	15	20	

Supplementary Table 1. Clinical characteristics of classified groups of TCGA colorectal cancer patients treated with 5-fluorouracil. Predicted responders and non-responders were classified using the expression levels of the "activation of BH3-only proteins" pathway. *P*-values were calculated using a chi-squared test. *P*-values < 0.05 were considered significant.

Variable		Cases	Predicted Response		
			Responder	NonResponder	P-value
Age (years)					
	<60	21	11	10	0.94
	≥60	56	28	28	
Gender					
	Male	53	27	26	0.87
	Female	24	12	12	
T stage					
	T1-T2	12	6	6	0.79
	T3-T4	65	33	32	
Lymph node stage					
	N0	39	22	17	0.093
	N1	13	3	10	
	N2	25	14	11	

Supplementary Table 2. Clinical characteristics of classified groups of TCGA bladder cancer patients treated with cisplatin. Predicted responders and non-responders were classified using the expression levels of the "amino acid synthesis and interconversion" pathway. *P*-values were calculated using a chi-squared test. *P*-values < 0.05 were considered significant.

Cancer	Drug	Pathway	Proximity (z)	Mean. Sensitive	Mean. Resistant	P-value
COAD	5FU	MITOTIC_G1_G1_S_PHASES	-4.26	0.34	0.31	0.15
		G1_S_TRANSITION	-3.9	0.38	0.34	0.15
		PYRIMIDINE_METABOLISM	-3.42	0.17	0.16	0.11
BLCA	Cisplatin	METAL_ION_SLC_TRANSPORTERS	-4.03	0.078	0.06	0.014
		RESPONSE_TO_ELEVATED_PLATELET_CYTOSOLIC_CA2_	-3.75	0.14	0.12	0.00054
		RECYCLING_OF_BILE_ACIDS_AND_SALTS	-3.73	-0.28	-0.22	0.01

Supplementary Table 3. Expression differences of the top three proximal pathways between drug-sensitive and -resistant isogenic cancer cell lines. The top three most proximal pathways to 5-fluorouracil and cisplatin treatment were used to measure expression level differences. Network proximity (z-scores), mean pathway expression levels in drug-sensitive and -resistant cancer cell lines, and *P*-values for expression level differences are displayed. A two-sample, two-tailed Student's t-test was used to calculate the *P*-values.

Cancer	Drug	Pathway	Proximity (*z*)
COAD	5FU	PI3K_EVENTS_IN_ERBB2_SIGNALING	1.4
BLCA	Cisplatin	REGULATION_OF_KIT_SIGNALING	-1.51
		NOTCH1_INTRACELLULAR_DOMAIN_REGULATES_TRANSCRIPTION	-0.47
		SIGNALING_BY_NOTCH1	-0.16
		ADAPTIVE_IMMUNE_SYSTEM	1.63
		IL_2_SIGNALING	2.09
		CA_DEPENDENT_EVENTS	2.94

Supplementary Table. 4. Network proximity for predictive pathways of drug response from bootstrapping analysis. Pathways that ranked equal to or higher than our predictions in the bootstrapping analysis are displayed.
